# Gorham-Stout case report: a multi-omic analysis reveals recurrent fusions as new potential drivers of the disease

**DOI:** 10.1186/s12920-022-01277-x

**Published:** 2022-06-06

**Authors:** Marcos Yébenes Mayordomo, Sofian Al Shboul, Maria Gómez-Herranz, Asim Azfer, Alison Meynert, Donald Salter, Larry Hayward, Anca Oniscu, James T. Patton, Ted Hupp, Mark J. Arends, Javier Antonio Alfaro

**Affiliations:** 1 International Center for Cancer Vaccine Science (ICCVS), University of Gdansk, Gdańsk, Poland; 2 Edinburgh Pathology, Institute of Genetics and Cancer (IGC), University of Edinburgh, Edinburgh, Scotland; 3 Department of Basic Medical Sciences, Faculty of Medicine, The Hashemite University, Zarqa, Jordan; 4 MRC Human Genetics Unit, MRC Institute of Genetics and Cancer, University of Edinburgh, Edinburgh, Scotland; 5 Department of Pathology, Royal Infirmary of Edinburgh, Edinburgh, Scotland; 6 Department of Orthopaedic Surgery, Royal Infirmary of Edinburgh, Edinburgh, Scotland

**Keywords:** Gorham-Stout, Genomics, Transcriptomics, Autophagy, Fusions, Mutations, *PI3K*, *AKT*, *mTOR*, Case report

## Abstract

**Background:**

Gorham-Stout disease is a rare condition characterized by vascular proliferation and the massive destruction of bone tissue. With less than 400 cases in the literature of Gorham-Stout syndrome, we performed a unique study combining whole-genome sequencing and RNA-Seq to probe the genomic features and differentially expressed pathways of a presented case, revealing new possible drivers and biomarkers of the disease.

**Case presentation:**

We present a case report of a white 45-year-old female patient with marked bone loss of the left humerus associated with vascular proliferation, diagnosed with Gorham-Stout disease. The analysis of whole-genome sequencing showed a dominance of large structural DNA rearrangements. Particularly, rearrangements in chromosomes seven, twelve, and twenty could contribute to the development of the disease, especially a gene fusion involving *ATG101* that could affect macroautophagy. The study of RNA-sequencing data from the patient uncovered the *PI3K*/*AKT*/*mTOR* pathway as the most affected signaling cascade in the Gorham-Stout lesional tissue. Furthermore, M2 macrophage infiltration was detected using immunohistochemical staining and confirmed by deconvolution of the RNA-seq expression data.

**Conclusions:**

The way that DNA and RNA aberrations lead to Gorham-Stout disease is poorly understood due to the limited number of studies focusing on this rare disease. Our study provides the first glimpse into this facet of the disease, exposing new possible therapeutic targets and facilitating the clinicopathological diagnosis of Gorham-Stout disease.

**Supplementary Information:**

The online version contains supplementary material available at 10.1186/s12920-022-01277-x.

## Background

Gorham-Stout disease (GSD) (OMIM #123880) or vanishing bone disease is an extremely rare illness that causes a proliferation of lymphatic vascular channels inducing massive osteolysis and bone loss. Less than 400 cases have been reported since the disease was first described in 1955 [1], leading to challenges in diagnosing and treating the disease. Although it can affect any part of the skeleton, the shoulders and pelvis are the most commonly affected areas [2].

The disorder can be diagnosed at any age but is generally present in patients between 13 and 30 years of age with no sex or ethnic predisposition [3]. The alterations in bone resorption may be one of the reasons why young adult patients are the main group affected by the disease [4], as resorption is part of normal bone growth. Main therapies consist of surgical excision and reconstruction of bone tissue, radiotherapy, and sirolimus (rapamycin) treatment. Rapamycin treatment represents a novel approach whose safety and efficacy remain unclear [5].

Recent research investigated mutations in this disorder, focusing on 50 cancer-related genes and revealing a somatic activating mutation in *KRAS* causing a gain of function [6]. This variant has been previously reported in other cancer studies [7, 8]. It is known to promote cell growth by activating the *RAS/MAPK* and *PI3K/AKT* signaling pathways relevant to lymphatic vascular growth and angiogenesis [9]. Concurrent with this study, *Nassim Homayun-Sepehr et al* [10] presented a model where a different mutation affecting *KRAS* also activates the development of lymphatic vessels in bone through the same signaling cascade.

The aim of our study is to provide new insights into the genomics and transcriptomics characteristics of the Gorham-Stout disease by performing the first multi-omics exploration of whole-genome sequencing data and RNA-sequencing data in a Gorham-Stout patient.

## Case presentation

The 45-year-old white female patient presented with left arm vascular proliferative disease within and around the humerus bone resulting in a pathological fracture. A clinical diagnosis of Gorham-Stout disease was made following pathological and radiological investigations of the lesion at this site (Additional file 1: Fig. S1). Macroscopic examination showed an abnormality of the upper arm muscles which appeared vascular and spongiotic, soft in some areas and fibrotic in others. The cortical bone of the humerus was thin around the fracture site and the bone marrow appeared, similar to the soft tissue, vascular with some large cysts and hemorrhage noted. Tissue blocks of all the abnormal areas from the soft tissue and humeral bone were sampled for histological examination and further investigations.

Histological sections of the affected regions confirmed indeed an abnormal vascular proliferation dissecting through fibroadipose connective tissue, skeletal muscle, and bone. Despite its dissecting nature, the vascular proliferation was composed of thin-walled vascular spaces and papillary projections lined by cytologically bland endothelial cells. The site of the fracture showed reparative fibrotic changes, with extensive granulation tissue and fibrosis. The dissecting vascular proliferation also affected the bone and was associated with cystic changes within the bone. The bone marrow showed vascular and fibrotic changes with some granulation tissue. Occasional discrete granulomas were scattered throughout the lesion. These changes are all consistent with the clinical diagnosis of Gorham-Stout disease or vanishing bone disease. Past medical history includes a previous diagnosis 1–2 years earlier of a benign vascular proliferation/vascular malformation involving both bone and soft tissue of the left humerus compatible with Gorham-Stout disease. The expert pathological review from the Department of Musculoskeletal Pathology, Royal Orthopaedic Hospital NHS Foundation Trust, Stanmore, was in agreement.

### Genomic exploration of Gorham-Stout (GS) lesional tissue

Small DNA variants were called using whole-genome sequencing data of GS vascular proliferation tissue and surrounding normal tissue extracted from the same patient (Additional file 2: Methods). Although the clinical characteristics of GS can be similar to those manifested in Ewing’s sarcoma [11], the genomic profile differs from most cancer-like diseases, as it doesn’t seem to be mutation-driven. A total of 643 mutations in 233 genes were found in GS lesional tissue when compared to the adjacent normal. Of those mutated genes, neither *TP53, RB1, CDKN2A* nor any of the known sarcoma biomarkers [12] were reported to contain any small mutations.

**Fig. 1:**
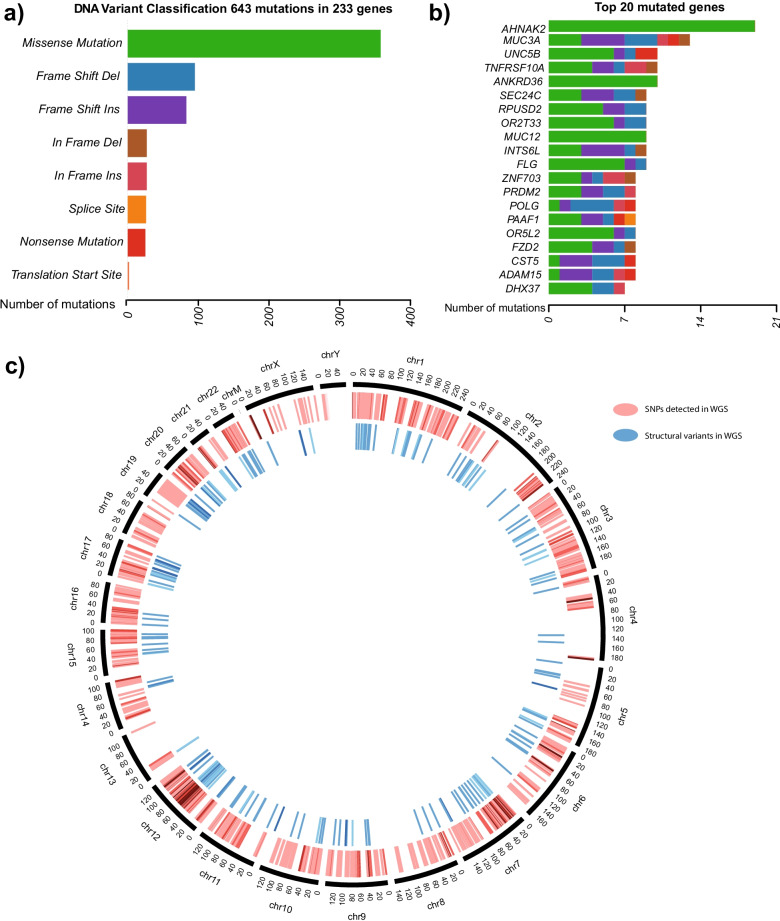
Gorham stout whole-genome sequencing mutation exploration. **a** 643 single nucleotide variants and small mutations were detected in 233 genes in the GS tissue, **b** Top 20 genes containing the most number of mutations in the GS tissue. **c** Circos plot comparing the number of small mutations (red) and structural variants (blue)

The classification of variants (Fig. 1a) showed that, although most of the variants code for missense mutations, a considerable number of insertions and deletions were found. Among the top mutated genes (Fig. 1b), most of the genes reported contained insertions, deletions, or splice site changes. Some known cancer-related genes like the mucin family (*MUC3A* and *MUC12*) in colorectal cancer, or *ZNF703* in breast cancer, seemed to be mutated. Other genes like *CST5* and *UNC5B*, related by previous studies to the *P53* pathway [13, 14], were also involved in other signaling pathways that are manifested in GS, like endochondral ossification and autophagy respectively. Other relevant gene families affected are those from *TNFRSF10A* and *ANKRD36* genes, previously identified as mutated in a scapular lesion affected with Gorham-Stout disease [3].

### Structural variants and gene fusions

**Fig. 2:**
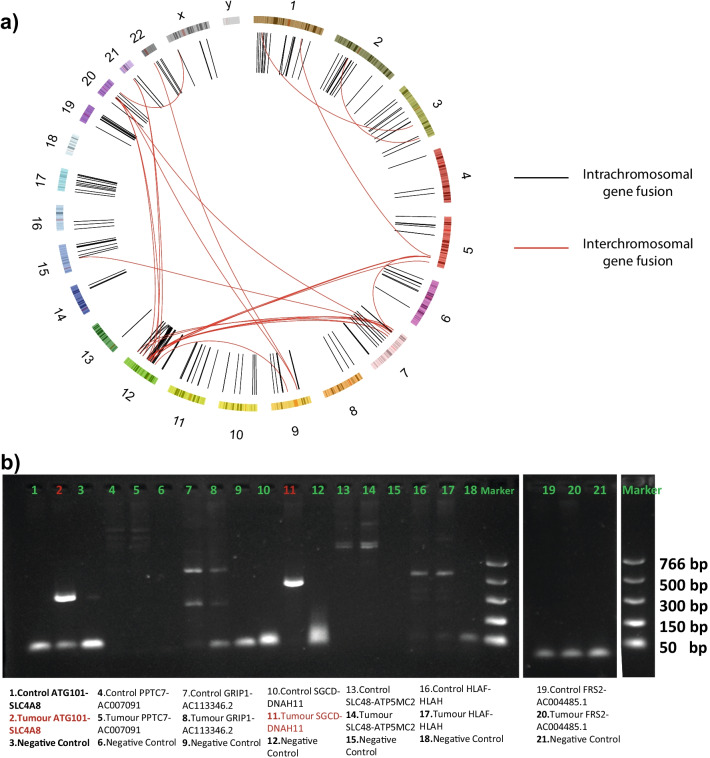
In silico study and validation of gene fusions detected in the GS tissue. **a** Circos plot of the gene fusions reveals a high number of interchromosomal fusions (red) especially affecting chromosomes 7, 12, and 20. **b** PCR Validation of the gene fusion candidates. Each fusion contains 3 columns showing the normal adjacent tissue DNA, the GS tissue DNA and a negative control from left to right respectively

We noticed a high proportion of genes with insertions and deletions displayed in the top 20 mutated genes. This motivated an analysis of larger indels and structural variants to explore the possibility that those alterations might have been caused by major chromosomal events. (Fig. 1c). Matching our previous discoveries, a high number of structural variants were identified, especially in chromosomes seven, twelve, and twenty. Around 1000 structural variants were found, showing duplications, deletions, and tandem repetitions. The most frequent structural events detected were chromosome translocations, suggesting that gene fusion variants could be a major event for GS disease. To further explore these rearrangements, we combined DNA and RNA gene fusion calls for the case (Fig. 2a). The fusions were categorized in different tiers depending on the evidence of the mutation at both the DNA and RNA level based on the genes surrounding the fusion or reads containing the translocation (Additional file 3: Table S1). Although most of the fusions reported were intrachromosomal events, chromosomes twelve, seven, and twenty shared a relevant number of interchromosomal mutations.

Based on the evidence of the gene fusions and their biological relevance, seven gene fusions were selected for RT-PCR validation (Additional file 4: Fig. S2). The fusion of *ATG101* and *SLC4A8* in chromosome 12 (Fig. 2b) involves the autophagy-related protein part of the macroautophagy signaling pathway [15]. The other validated fusion (Fig. 2b) involves sarcoglycan delta (*SGCD*), a gene known to cause muscular dystrophy in mammals when deleted or mutated [16–19], an event that could lead to vascular malformations as reported in the GS lesions.

### Gene expression in Gorham-Stout disease

**Fig. 3:**
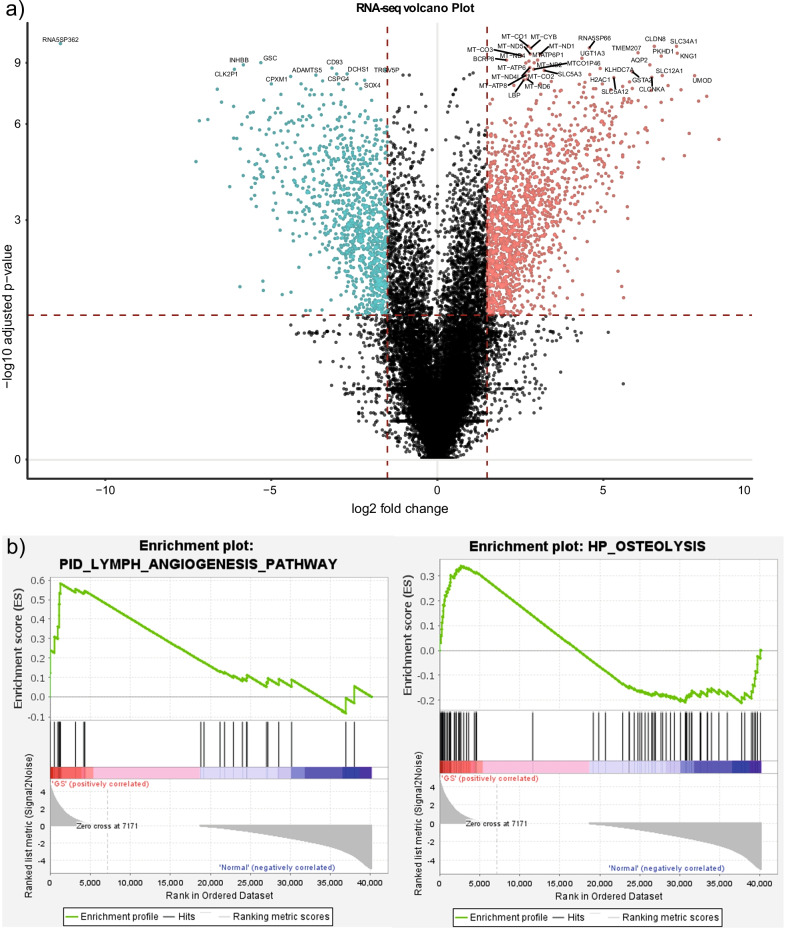
RNA expression analysis between Gorham-Stout tissue and adjacent normal. **a** Volcano plot of the RNA-seq differential expression analysis showing a large number of genes up and down-regulated in GS tissue. **b** Results of the gene set enrichment analysis (GSEA) selecting the lymph-angiogenesis (VEGF) and osteolysis (NOTCH) pathways. A heavily weighted distribution in multiple genes was found in both pathways, with a high number of genes overexpressed in either GS tissue or the attached normal

The differential expression analysis of the technical replicates from Gorham-Stout lesional tissue and attached normal revealed that a high proportion of genes (36.6% out of the 58,884 total) were either up-regulated or down-regulated (Fig. 3a). Gene set enrichment analysis showed statistically significant differences in pathways like lymphangiogenesis (Additional file 5: Table S2) and osteolysis (Additional file 5: Table S3), suspected of being drivers of the pathological characteristics and potential targets of drug treatments for the disease [20] (Fig. 3b). Inside these pathways, gene families like *VEGF* or *NOTCH* were detected to change drastically in expression from normal to Gorham-Stout lesional tissue (Additional file 7: Fig. S3).

One of the main pathways affected by changes in expression is the phosphatidylinositol 3-kinase (*PI3K*), involved in the proliferation, growth, and regulatory processes of the cell. In our study, we detected that the expression of *PI3K* is considerably downregulated in GS lesions when compared to normal tissue, therefore the phosphorylation of *PIP2* to *PIP3* by this gene will be decreased in the disease. This event is confirmed by the up-regulation of PTEN which regulates *PI3K* by the dephosphorylation of the *PIP3* product [21].

**Fig. 4:**
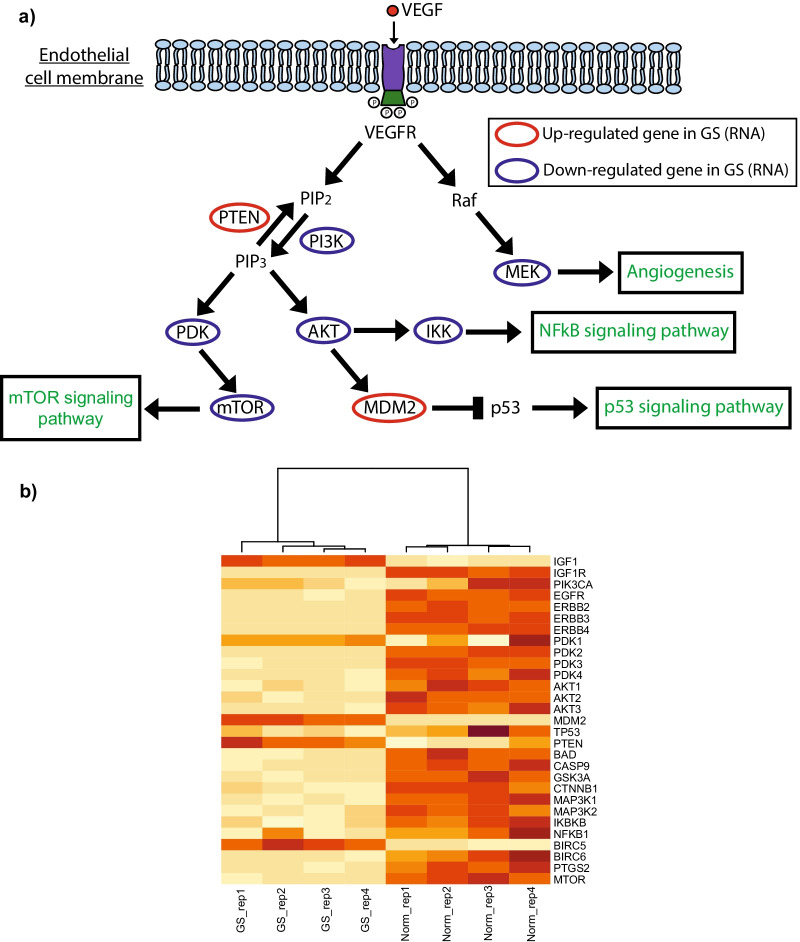
Alterations in the PI3K/AKT signaling pathway in GS tissue. **a** Brief representation of gene members in the PI3K/AKT pathway. Most genes showed changes in expression when comparing adjacent normal to GS tissue affecting other metabolic pathways. **b** Heatmap representation of the gene members affected by RNA-seq expression changes between normal and GS tissue. PTEN and MDM2 showed over-expression in GS tissue while most of the other affected genes appear down-regulated when compared to adjacent normal

The changes in expression of the *PI3K* and *PTEN* pathway (Fig. 4a–b) are reminiscent of other cancers, where the pathway is deactivated or mutated affecting regulation of mTOR [22]. The expression of *PI3K, AKT,* and *mTOR* in the Gorham-Stout lesion was decreased, while *PTEN* expression was higher when compared to the attached normal. The alterations suggest the promotion of irregular endothelial cell growth and angiogenesis through *VEGFA* and *VEGFB* via the *VEGFR1-PI3K-AKT* signaling pathway [23, 24].

Another expression event that may be related to cancer is the activation of the *NF-kB* signaling pathway, which is down-regulated in Gorham-Stout lesional tissue when compared to matched normal. The levels of expression of *NF-kB* and *IKK* in the normal adjacent tissue could contribute to inflammation and macrophage activation [25, 26].

Although the expression changes previously mentioned are occurring in normal adjacent tissue, there are events in GS tissue that share similarities with cancer. One of them is the high expression of *MDM2*, a p53-specific E3 ubiquitin ligase, which leads to the degradation of the *p53* tumor suppressor protein [27]. All the events linked to the *PI3K* signaling pathway paint a picture of the possible inner mechanisms in Gorham-Stout and surrounding tissue providing unique insights into the disease that could lead to the development of new therapeutic strategies targeting the mentioned pathways.

### Immune infiltrates in Gorham-Stout lesional tissue

**Fig. 5:**
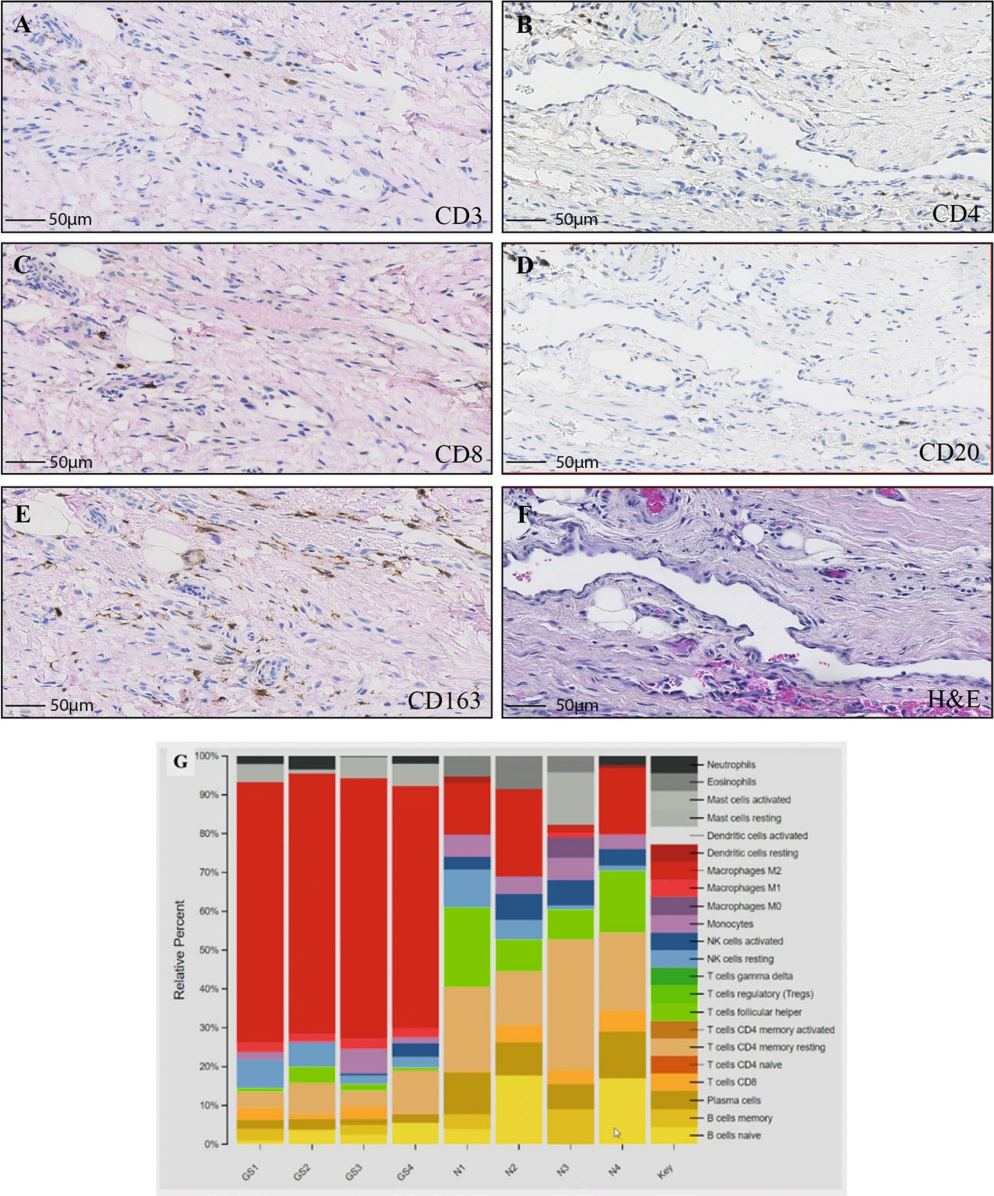
Representative IHC stained images showing the distribution of CD3, CD4, CD8, CD20, and CD163 cell markers. The representative images exhibit the immunohistochemical features of infiltrating immune cells: **a** CD3^+^ T cells, **b** CD4^+^ T cells, **c** CD8^+^ T cells, **d** CD20^+^ B cells, **e** CD163^+^ M2 macrophages, and **f** H&E staining. Scale bars show 50 μm. **g** RNA comparison of a variety of immune cell types between Gorham-Stout disease and normal specimens. The analysis was conducted with 4 technical replicates

 Immunohistochemical and H&E staining was carried out using formalin-fixed, paraffin-embedded (FFPE) sections to investigate the immune system response of a single case. Evaluation of the immune cell infiltration was assessed by immunohistochemistry with five immune cell markers: CD3^+^ T cells, CD4^+^ T cells, CD8^+^ T cells, CD20^+^ B cells, and CD163^+^ M2 macrophages (Fig. 5A–F). Stained Gorham-Stout disease slides were scanned using a Hamamatsu NanoZoomer XR slide scanner at × 40 magnification. Digital images were analyzed using QuPath (version 0.2.0-m7) to quantify the positive staining, validated by manual counting in selected areas that showed a highly significant correlation (Table 1).

**Table 1:** Summary of the percentage of cells stained for CD3, CD4, CD8, CD20, and CD163 cell markers within the Gorham-Stout disease sample

Sample	Positive (%)	Rho	P value
CD3	8.22	0.997	< 0.0001
CD4	0.06	0.986	< 0.0001
CD8	5.93	0.991	< 0.0001
CD20	4.86	0.995	< 0.0001
CD163	20.01	0.996	< 0.0001

The biopsy sections were stained with the following cell markers: CD3, CD4, CD8, CD20, and CD163. Positively stained cells (expressed as a percentage of total cells) were automatically counted using QuPath (version 0.2.0-m7). The methodology was verified by comparing manual counting with QuPath counting in 0.2 mm. [44–46] areas selected randomly across the different sections. Pearson’s correlation (Rho) and P values were calculated

The Gorham-Stout disease specimen showed significantly higher CD163^+^ M2 macrophage infiltration, compared with other examined immune cell markers (Fig. 5E and Table 1). This was consistent with the RNA data that revealed increased infiltration of M2 macrophages in Gorham-Stout disease compared with normal samples (Fig. 5G). In contrast, CD4^+^ T cell staining was particularly low with only 0.06% positive staining (Fig. 5B and Table 1). Moderate staining was found for the other 4 lymphocytic cell markers: CD3^+^ T cells (8.22%), CD8^+^ T cells (5.93%), and CD20^+^ B cells (4.86%).

## Discussion and conclusions

Large chromosomal events were detected in chromosomes seven, twelve, and twenty, where gene fusions were the dominant event. The gene fusion of *ATG101* and *SLC4A8* (Additional file 8: Fig. S4) involved a binding protein (*ATG101*) essential for macroautophagy [15, 28], which could affect the macrophag e signaling pathway. This event has to be confirmed in other Gorham-Stout patients, as the study of the structural variants and gene fusions is a novel insight in this field. Evidence for this fusion to be pathological was strengthened upon further investigation by ensuring adherence to the ACMG standards and guidelines for the interpretation of sequence variants [29] (Additional file 2: Methods).

The small mutations found in this case did not match any of the previously mutated genes found in the literature, although genes were belonging to the same families and/or affected the same pathways previously found in neoplasms. The tumor necrosis factor receptor *TNFRSF10A* was detected in our study among the top five mutated genes showing multiple deletions, insertions, and point mutations. *TNFRSF11A*, a member of the same family of genes, was reported in a previous case report of a Gorham-Stout patient [3] and linked to muscular dystrophy and osteolysis [30, 31]. Another example of mutated gene families affecting the same pathway is the missense mutation found in *PIK3AP1* (c.1139A > T), which belongs to the *PTEN/PI3K/AKT* signaling cascade. Genes belonging to this family, like *PIK3CA*, are known to cause lymphatic and vascular overgrowth disorders [32] while others like *PTEN* have been reported as mutated in Gorham-Stout disease patients [33].

The alterations of the *PTEN/PI3K/AKT* signaling cascade were not only observed by mutations of some of the members of the pathway but also reported as gene expression changes in the RNA sequencing data. We observed that most of the genes involved in the signaling cascade were either up-regulated or down-regulated when compared to normal adjacent tissue. The modifications of the PI3K pathway are known to cause lymphatic malformations [9, 34] and during recent years have become the main target for inhibitor therapies designed to decrease *VEGF* secretion and angiogenesis [35–38]. Although the *PI3K* pathway was already known to be affected in Gorham-Stout disease, as well as other lymphatic malformations, our study is the first to profile Gorham-Stout lesions by RNA-Seq analysis and this has demonstrated new possible candidates for therapy like the targeting of *MDM2-p53* already developed for cancer therapy [39, 40].

Besides angiogenesis and osteolysis, another characteristic of GS disease is osteoclast formation. Previous studies have suggested this event is stimulated by macrophage secretion of *TNFα* and *IL-6* [41] and linked it to the clinical characteristics of the disease [42]. Our study has shown that M2 macrophages tend to infiltrate the Gorham-Stout vascular proliferation tissue, while other immune cells appear to be less frequent. The results match previous findings in the literature where CD163 staining was also performed [6], as well as the mostly negative staining for other immune cells [43].

We have presented a detailed molecular investigation of a single patient with Gorham-Stout disease. Whole-genome sequencing data of the Gorham-Stout vascular proliferation lesion revealed that the main driver of the genomic events appears to be large structural alterations, though single nucleotide variants and small mutations were also present. The transcriptomics showed changes in expression between the normal and the Gorham-Stout tissue, involving the osteolysis and angiogenesis pathways. The alteration of the *PI3K/AKT/mTOR* pathway along with the macrophage infiltration in the Gorham-Stout tissue are congruent with emerging trends in this disease. As with any rare disease, the inclusion of further GSD patients into the future with a combined genomic and transcriptomic profile could confirm the insights we have revealed on the mechanisms of the disease.

## Supplementary Information

**Additional file1: Figure S1.** Pathological fracture of left humerus caused by Gorham-Stout disease in a 45-year-old white female patient. Sequential radiographs over a 1-year period show gradual disappearance of the proximal humerus.

**Additional file 2. Supplementary Methods.** Detailed information about sample processing for DNA, RNA sequencing and IHC analysis.

**Additional file3: Table S1.** Table of gene fusions detected and selected for validation including chromosome information and the number of reads in DNA and RNA level.

**Additional file4: Figure S2.** Gel electrophoresis containing control, Gorham-Stout tissue, and negative control of the gene fusion candidates. In the second gel only one set of amplified products was used in the final photograph as 19.Control FRS2-AC004485.1, 20.GS-FRS2-AC004485.1 21.Negative PCR Control (FRS2-AC004485.1). The wells 19, 20, and 21 were cropped in the final photograph including the PCR marker. A quick load PCR marker was used from NEB #N0475.

**Additional file5: Table S2.** Table results of the gene set expression analysis for lymphangiogenesis showing the affected genes in the RNA-seq expression and their contribution to the pathway.

**Additional file6: Table S3.** Table results of the gene set expression analysis for osteolysis showing the affected genes in the RNA-seq expression and their contribution to the pathway.

**Additional file7: Figure S3.** Barplot of RNA-seq expression (TPM) of VEGFC/D and NOTCH 2/3/4 genes in Gorham-Stout tissue compared with adjacent normal.

**Additional file8: Figure S4.** Gene fusion between ATG101 and SLC4A8 detected in Gorham-Stout patient. RNA evidence reads: 118 encompasing and 138 spanning reads. DNA evidence reads: 411 encompassing and 139 spanning reads in Gorham-Stout tissue. 0 reads found in normal tissue.

## Data Availability

The raw datasets generated and analyzed during the current study are not publicly available in order to protect participant confidentiality. The datasets obtained during the current study are available from the corresponding author if the requirements are reasonable.

## Abbreviations

GSD: Gorham-Stout disease
GS: Gorham-Stout
WGS: Whole-genome sequencing
BWA: Burrow-Wheeler aligner
Hg38: Homo sapiens genome assembly GRCh38
GATK: Genome Analysis Toolkit
SNV: Single nucleotide variants
Indel: Insertions and deletions
RSEM: RNA-seq by expectation maximization
TPM: Transcripts per million
DEA: Differential expression analysis
IHC: Immunohistochemistry
H&E: Hematoxylin and eosin
FFPE: Formalin-fixed, paraffin-embedded
SGCD: Sarcoglycan delta
PI3K: Phosphatidylinositol 3-kinase

## References

[1] Gorham LW, Stout AP (1955). Massive osteolysis (acute spontaneous absorption of bone, phantom bone, disappearing bone); its relation to hemangiomatosis. J Bone Joint Surg Am.

[2] Patel DV (2005). Gorham’s disease or massive osteolysis. Clin Med Res.

[3] Li M (2018). Successful management of Gorham-Stout disease in scapula and ribs: a case report and literature review. Orthop Surg.

[4] Yerganyan VV, Body JJ, De Saint-Aubain N, Gebhart M (2015). Gorham-Stout disease of the proximal fibula treated with radiotherapy and zoledronic acid. J Bone Oncol.

[5] Liang Y (2020). Gorham-Stout disease successfully treated with sirolimus (rapamycin): a case report and review of the literature. BMC Musculoskelet Disord.

[6] Nozawa A (2020). A somatic activating KRAS variant identified in an affected lesion of a patient with Gorham-Stout disease. J Hum Genet.

[7] Aoki Y, Niihori T, Inoue S, Matsubara Y (2016). Recent advances in RASopathies. J Hum Genet.

[8] Muñoz-Maldonado C, Zimmer Y, Medová M (2019). A comparative analysis of individual ras mutations in cancer biology. Front Oncol.

[9] Nguyen H-L, Boon LM, Vikkula M (2017). Vascular anomalies caused by abnormal signaling within endothelial cells: targets for novel therapies. Semin Interv Radiol.

[10] Homayun-Sepehr N (2021). *KRAS*-driven model of Gorham-Stout disease effectively treated with trametinib. JCI Insight.

[11] Zheng C (2019). Gorham-Stout disease of the malleolus: a rare case report. BMC Musculoskelet Disord.

[12] Steele CD (2019). Undifferentiated sarcomas develop through distinct evolutionary pathways. Cancer Cell.

[13] Hünten S, Hermeking H (2015). p53 directly activates cystatin D/CST5 to mediate mesenchymal-epithelial transition: a possible link to tumor suppression by vitamin D3. Oncotarget.

[14] Yang Z (2021). UNC5B promotes vascular endothelial cell senescence via the ROS-mediated P53 pathway. Oxid Med Cell Longev.

[15] Mercer CA, Kaliappan A, Dennis PB (2009). A novel, human Atg13 binding protein, Atg101, interacts with ULK1 and is essential for macroautophagy. Autophagy.

[16] Durbeej M, Campbell KP (2002). Muscular dystrophies involving the dystrophin-glycoprotein complex: an overview of current mouse models. Curr Opin Genet Dev.

[17] Cox ML (2017). Exome sequencing reveals independent SGCD deletions causing limb girdle muscular dystrophy in Boston terriers. Skelet Muscle.

[18] Townsend D (2014). Finding the sweet spot: assembly and glycosylation of the dystrophin-associated glycoprotein complex. Anat Rec.

[19] Younus M (2018). SGCD homozygous nonsense mutation (p.Arg97(∗)) causing limb-girdle muscular dystrophy type 2F (LGMD2F) in a consanguineous family: a case report. Front Genet.

[20] Mavrogenis AF, Zambirinis CP, Dimitriadis PA, Tsakanikas A, Papagelopoulos PJ (2010). Gorham-stout disease. J Surg Orthop Adv.

[21] Carracedo A, Pandolfi PP (2008). The PTEN–PI3K pathway: of feedbacks and cross-talks. Oncogene.

[22] Chalhoub N, Baker SJ (2009). PTEN and the PI3-kinase pathway in cancer. Annu Rev Pathol.

[23] Karar J, Maity A (2011). PI3K/AKT/mTOR pathway in angiogenesis. Front Mol Neurosci.

[24] Pandey AK (2018). Mechanisms of VEGF (vascular endothelial growth factor) inhibitor-associated hypertension and vascular disease. Hypertension.

[25] Liu T, Zhang L, Joo D, Sun S-C (2017). NF-κB signaling in inflammation. Signal Transduct Target Ther.

[26] Timmer AM, Nizet V (2008). IKKβ/NF-κB and the miscreant macrophage. J Exp Med.

[27] Moll UM, Petrenko O (2003). The MDM2-p53 interaction. Mol Cancer Res.

[28] Hosokawa N (2009). Atg101, a novel mammalian autophagy protein interacting with Atg13. Autophagy.

[29] Richards S (2015). Standards and guidelines for the interpretation of sequence variants: a joint consensus recommendation of the American College of Medical Genetics and Genomics and the Association for Molecular Pathology. Genet Med.

[30] Passarelli C (2020). Tumor necrosis factor receptor SF10A (TNFRSF10A) SNPs correlate with corticosteroid response in duchenne muscular dystrophy. Front Genet.

[31] Hughes AE (2000). Mutations in TNFRSF11A, affecting the signal peptide of RANK, cause familial expansile osteolysis. Nat Genet.

[32] Luks VL (2015). Lymphatic and other vascular malformative/overgrowth disorders are caused by somatic mutations in PIK3CA. J Pediatr.

[33] Hopman SMJ (2012). PTEN hamartoma tumor syndrome and Gorham-Stout phenomenon. Am J Med Genet A.

[34] Ozeki M, Fukao T (2019). Generalized lymphatic anomaly and gorham-stout disease: overview and recent insights. Adv Wound Care.

[35] Mathew M, Goyal A, Khan A, Yuen T, Zaidi M (2020). Drugs for rare diseases of bone. Encyclopedia of bone biology.

[36] Rössler J, Saueressig U, Kayser G, von Winterfeld M, Klement GL (2015). Personalized therapy for generalized lymphatic anomaly/gorham-stout disease with a combination of Sunitinib and Taxol. J Pediatr Hematol Oncol.

[37] Hammer F (2005). Gorham-Stout disease-stabilization during bisphosphonate treatment. J Bone Miner Res Off J Am Soc Bone Miner Res.

[38] Nozawa A (2016). Gorham-stout disease of the skull base with hearing loss: dramatic recovery and antiangiogenic therapy. Pediatr Blood Cancer.

[39] Shangary S, Wang S (2008). Targeting the MDM2-p53 interaction for cancer therapy. Clin Cancer Res Off J Am Assoc Cancer Res.

[40] Chène P (2003). Inhibiting the p53–MDM2 interaction: an important target for cancer therapy. Nat Rev Cancer.

[41] Faruqi T (2014). Molecular, phenotypic aspects and therapeutic horizons of rare genetic bone disorders. BioMed Res Int.

[42] Yeter HH (2017). Gorham-Stout disease or new entity on the basis of vasculopathy. Alex J Med.

[43] Colucci S (2006). Gorham-stout syndrome: a monocyte-mediated cytokine propelled disease. J Bone Miner Res.

[44] Bankhead P, Loughrey MB, Fernández JA, Dombrowski Y, McArt DG, Dunne PD, McQuaid S, Gray RT, Murray LJ, Coleman HG, James JA, Salto-Tellez M, Hamilton PW. QuPath: Open source software for digital pathology image analysis. Sci Rep. 2017. 10.1038/s41598-017-17204-5. 10.1038/s41598-017-17204-5 PMC5715110 29203879

[45] Berben L, Wildiers H, Marcelis L, Antoranz A, Bosisio F, Hatse S, Floris G (2020). Computerised scoring protocol for identification and quantification of different immune cell populations in breast tumour regions by the use of QuPath software. Histopathology.

[46] Al Shboul S, Curran OE, Alfaro JA, Lickiss F, Nita E, Kowalski J, Naji F, Nenutil R, Ball KL, Krejcir R, Vojtesek B, Hupp TR, Brennan PM (2021). Kinomics platform using GBM tissue identifies BTK as being associated with higher patient survival. Life Sci Alliance.

