# Supplementary material for: Gorham-Stout case report: a multi-omic analysis reveals recurrent fusions as new potential drivers of the disease
Source: BMC Med Genomics. 2022 Jun 6;15:128. doi: 10.1186/s12920-022-01277-x (PMC9169400; doi:10.1186/s12920-022-01277-x)
Supplement: Supplementary file 1 — Additional file1: Figure S1. Pathological fracture of left humerus caused by Gorham-Stout disease in a 45-year-old white female patient. Sequential radiographs over a 1-year period show gradual disappearance of the proximal humerus. [file 12920_2022_1277_MOESM1_ESM.pdf]

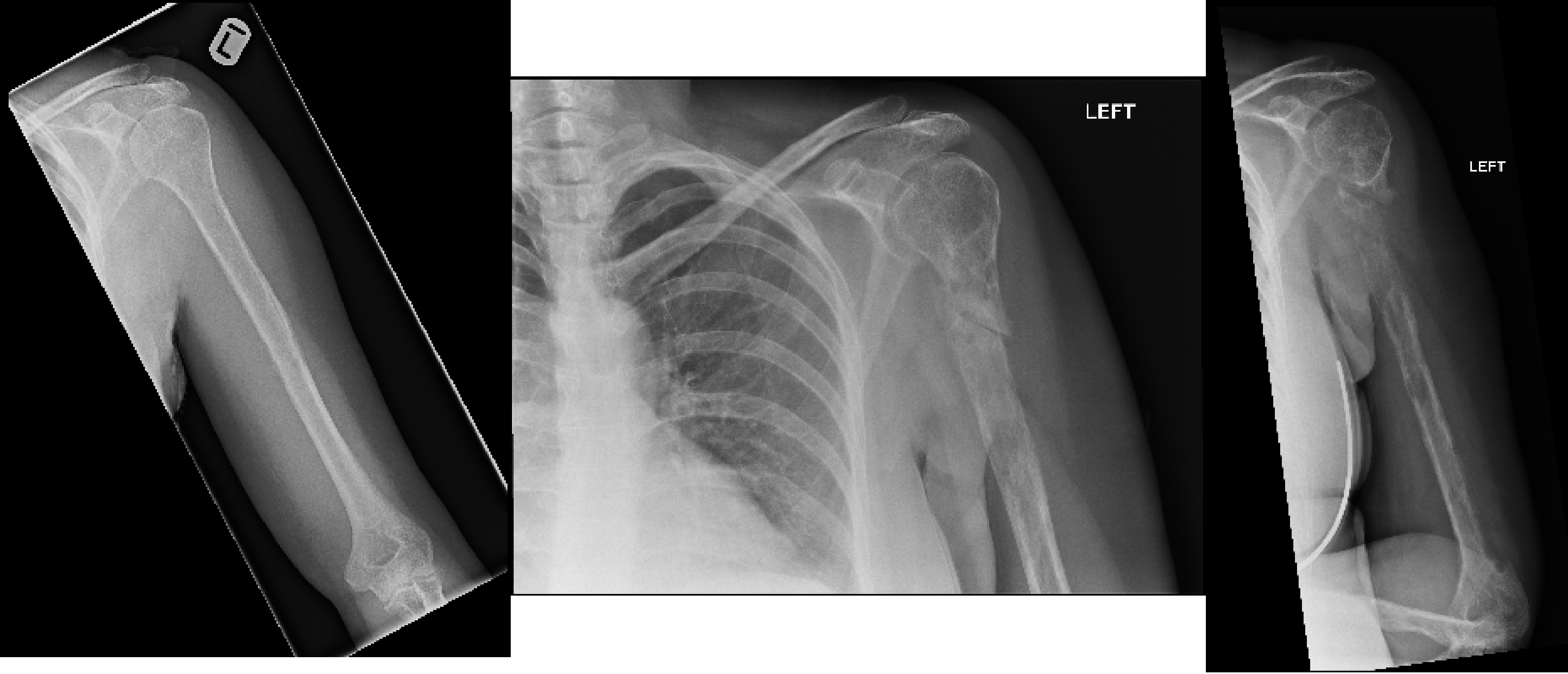

Supplementary Figure 1. Pathological fracture of left humerus caused by Gorham-Stout disease in a 45-year-old white female patient. Sequential radiographs over a 1-year period show gradual disappearance of the proximal humerus.
