# Supplementary material for: Gorham-Stout case report: a multi-omic analysis reveals recurrent fusions as new potential drivers of the disease
Source: BMC Med Genomics. 2022 Jun 6;15:128. doi: 10.1186/s12920-022-01277-x (PMC9169400; doi:10.1186/s12920-022-01277-x)
Supplement: Supplementary file 2 — Additional file 2. Supplementary Methods. Detailed information about sample processing for DNA, RNA sequencing and IHC analysis. [file 12920_2022_1277_MOESM2_ESM.docx]

# Supplementary methods

## Sample collection

Tissue was donated to the Lithian NRS BioResource under the patient’s consent and stored by The Tissue Governance Manager following the general approval for use of surgically obtained tissue established by NHS Lothian and the Public Health Office.

## DNA sequencing

Edinburgh Genomics Clinical utilizes Illumina SeqLab, which integrates Illumina TruSeq library preparation, Illumina cBot2 cluster generation, Illumina HiSeqX sequencing, Hamilton Microlab STAR integrative automation, and Genologics Clarity LIMS X Edition.

**Sample QC.** Genomic DNA (gDNA) samples are evaluated for quantity using Quant-iT Picogreen reagent, Lambda Standard DNA and a Molecular Devices, Spectramax XPS Gemini plate reader. The quality of the gDNA samples is evaluated using an AATI Fragment Analyzer and the Standard Sensitivity Genomic DNA Analysis Kit. Genomic DNA samples were found to have a total amount of >-1000ng and a quality score >-5 pass sample QC. Based on the quantification results, gDNA samples are pre-normalized to fall within the 5-40ng=µl concentration range required for Illumina SeqLab TruSeq Nano library preparation method using the Hamilton MicroLab STAR.

**Library Preparation.** Next-Generation sequencing libraries are prepared using Illumina SeqLab specific TruSeq PCRFree High Throughput library preparation kits in conjunction with the Hamilton MicroLab STAR and Clarity LIMS X Edition. The gDNA samples are normalized to the concentration and volume required for the Illumina TruSeq PCR-Free library preparation kits then sheared to a 450bp mean insert size using a Covaris LE220 focused-ultrasonicator. The inserts are blunt-ended, A-tailed, size selected and the TruSeq adapters are ligated onto the ends.

**Library QC.** The insert size for each library is evaluated using the Caliper GX Touch with an HT DNA 1k/12K/HI SENS LabChip and HT DNA HI SENS Reagent Kit to ensure that the mean fragment sizes fall between 300bp and 800bp. The concentration of each library is calculated using a Roche LightCycler 480 and a Kapa Illumina Library Quantification kit and Standards to ensure that the concentration of each library is between 1.1nmol and 8nmol.

**Sequencing.** The libraries are normalized to 1.5nmol and are denatured for clustering and sequencing at 300pmol using a Hamilton MicroLab STAR with Genologics Clarity LIMS X (4.2) Edition. Libraries are clustered onto a HiSeqX Flow cell v2.5 on cBot2s and the clustered flow cell is transferred to a HiSeqX for sequencing using a HiSeqX Ten Reagent kit v2.5.

**WGS data analysis**. The data generated by Edinburgh Genomics with coverage of 30X for the normal and 60X for tumor samples was transferred in the form of raw sequencing reads (gzipped FASTQ file format). Alignment was performed using BWA mem (version 0.7.17) using Homo sapiens genome assembly GRCh38 (hg38) as a reference genome. Output SAM files were sorted, and converted to BAM format, and duplicates were marked using Picard (version 2.18.23). Base recalibration was performed using the Genome Analysis Toolkit (GATK version 4.1.3.0). Somatic short variants (SNVs and Indels) and copy number alterations were identified using GATK best practices workflow (version 4.1.3.0). Structural variants were discovered using the Illumina software called Manta Structural Variant Caller (version 1.6.0). The visualization of structural variants was performed using Circos (Galaxy version 0.69.8). Somatic short variants (SNVs and Indels) and copy number alterations were identified following the GATK best practices workflow, using HaplotypeCaller (version 4.1.3.0).

## RNA sequencing

**RNA-seq data analysis.** Normal and GSD samples were sent for RNA sequencing to the Genetics Core of the Edinburgh Clinical Research Facility. FASTQ files were aligned to GRCh38 using both TopHat (version 2.1.1) and STAR (version 2.6.1). The exploration of gene fusions was first performed on RNA-seq data only, using STAR-fusion. To validate the previous results and to add WGS data to this analysis, Integrate (version 0.2.6) was used to get a clearer insight into the gene fusions using both DNA and RNA sequencing data using the BAM files originated in the analysis. The evaluation of gene expression from GSD and normal samples were performed using RSEM normalizing the results using TPM values. Afterward, differential expression analysis (DEA) of the technical replicates was developed in R (version 3.6.3) using the Limma package (version 3.42.2).

**Gene set enrichment analysis** was performed using GSEA (version 4.1.0) with a ranked list of genes from the DEA data created in the RNA-seq analysis. The searches were performed combining different gene sets, of which we included the hallmark signature database, commonly used as an initial evaluation of the enrichment pathways, and further gene sets of lymphangiogenesis and osteolysis, which were suspected to be involved in the Gorham-Stout disease.

**RNA-seq deconvolution of the immune infiltrate.** The RNA-seq data were analyzed using CIBERSORTx, a tool that estimates the immune cell types of a mixed population. An expression matrix of the Gorham-Stout biopsy and the normal adjacent tissue was uploaded to the CIBERSORTx web server, processed, and compared to the expression matrix LM22, consisting of the profiling of 22 functionally defined human immune cell types[^1^](https://www.zotero.org/google-docs/?18P2Zq).

## Immunohistochemistry

Immunohistochemistry (IHC) staining was performed in the Pathology Department at the University of Edinburgh. Paraffin-embedded sections (5 μm thickness) were mounted on positively charged slides (Thermo Scientific) and stained using BOND III autostainer with Bond Polymer Refine Detection Kit (Leica Biosystems, DS9800) according to the manufacturer’s instructions. Briefly, slides were blocked with 4% hydrogen peroxide followed by antigen retrieval using Bond Epitope Retrieval Solution 1 (Citrate; pH 6.0) (Leica Biosystems, AR9961). Subsequently, the slides were incubated at room temperature with a primary antibody (**Table 1**) for 20 min. DAB (3,3′-diaminobenzidine) and hematoxylin counterstaining were used for visualization. Slides were dehydrated and cleared in xylene before coverslips were applied. Tonsil FFPE tissue blocks were used to optimize the antibody dilutions before applying to the examined sections.

Stained Gorham-Stout disease slides were scanned using a Hamamatsu NanoZoomer XR slide scanner at x40 magnification. Digital images were analyzed using QuPath, an open-source pathology and bioimaging software (version 0.2.0-m7)[^2^](https://www.zotero.org/google-docs/?UeHwze).

Gorham-Stout disease sections were examined manually to exclude artifacts or lack of material and all cells were detected using QuPath’s built-in cell segmentation algorithm, watershed cell detection command. For automated stained cell counting, the detection classifier was built using a random trees algorithm with the usage of 41 detection features. For building a functional detection classifier, cell annotations describing three types of cells were manually drawn. The first class identifies all DAB-stained and non-stained cells, the second class represents the cells that were mistakenly identified as DAB-positive cells (such as partially stained cells), and the third class detects artifacts. Subsequently, we set a cut-off point based on the staining intensity that distinguishes positive from negative cells. Each cell marker was separately optimized and quantified.

To assess the reliability of the automated counting using QuPath, 10 selected areas of 0.2 mm^2^ each were chosen across every section for manual counting. Differences between the manual and the automated counting were calculated using the following formula: (QuPath-manual)/manual. Differences were kept below 20%, the accepted interobserver variability[^3,4^](https://www.zotero.org/google-docs/?PUob8E). Pearson correlation between manual and automatic counting was calculated and p-values are two-sided with the significance threshold set at 0.05 (**Table 2**).

## PCR validation

DNA from the normal and GS tissues were extracted in a lysis buffer (50mM Tris-HCl, 100mM EDTA, 1% SDS, pH8.0) with overnight digestion by proteinase K (0.5mg/ml) at 56 ºC. The DNA was further processed by phenol/chloroform/isoamylalcohol (25:24:1). Finally, the DNA was precipitated by sodium acetate and 100% Alcohol and air-dried at room temperature. The DNA was re-suspended in 30-50 µl of water or TE.

A 2 μl aliquot of the DNA was amplified PCR reagent (Qiagen) in a thermal cycler at 95 °C for 3 min initial denaturation. 35 cycles of amplification were completed at 95 °C for 40 sec, annealing at 60 °C for 45 sec and extension at 72 °C for 1 min. PCR samples were resolved in 1.5% agarose gel in 0.5X TBE running buffer.

Taq DNA Polymerase kit was used from Qiagen PCR. The reagent and PCR steps were taken following **Table 3 and Table 4**. A 50 ng DNA from both tissues was used for amplification.

## ACMG standards for the interpretation of sequence variants

Variant calling was performed using 3 different computational software. Small nucleotide variants, insertions, and deletions were detected using the GATK best practices, using Mutect2 as a variant caller. For gene fusions, a combination of STAR-fusion and integrate was used as variant calling methods.

The gene fusion between *ATG101* and *SLC4A8* involves a disruption of ATG101 in chromosome 12 causing a truncated protein product due to the deletion of multiple amino acids (Clinvar ID: SUB11308769). The ACMG guidelines categorize this type of fusions as PM4 BP3. The use of multiple predictive software presenting evidence of a deletion in the gene/gene product (PP3) creates supporting evidence for the pathogenic role of the mutation. The qPCR validation of the gene fusions is a well-established functional study of the gene product adding strong evidence of pathogenicity (PS3).

Other criteria of the ACMG guidelines such as population data or segregation data were not applicable due to the individual analysis of the case report.

**Table 1 - Antibody details.** Concentration source and supplier of the antibodies used for immunohistochemistry.

**Table 2 - Summary of the percentage of cells stained for CD3, CD4, CD8, CD20, and CD163 cell markers within the Gorham-Stout disease sample.** The biopsy sections were stained with the following cell markers: CD3, CD4, CD8, CD20, and CD163. Positively stained cells (expressed as a percentage of total cells) were automatically counted using QuPath (version 0.2.0-m7). The methodology was verified by comparing manual counting with QuPath counting in 0.2 mm^2^ areas selected randomly across the different sections. Pearson's correlation (Rho) and p-values were calculated.

**Tables 3 and 4.** Protocol details of PCR reagent and steps followed for the validation.

**Table 1**

**
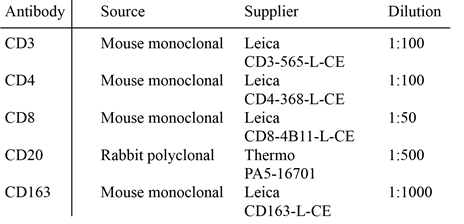
**

**Table 2**

**
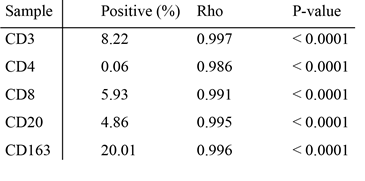
**

**Table 3**

|  | **x1(ul)** |
| --- | --- |
| Water | 12.375 |
| 10x Buffer | 2.5 |
| dNTP’s (10mM) | 1 |
| Q solution | 5 |
| Forward Primer | 1 |
| Reverse Primer | 1 |
| Taq | 0.125 |
| Template | 2 ul |
| **Total** | **25** |

**Table 4**

| **Step** | **Temp** | **Duration** |
| --- | --- | --- |
| Denature  Template | 94 | 3 min |
| Denature  Template | 94 | 40 sec |
| Anneal  primers | 65 | 45 sec |
| Extend  primers | 72 | 1 min |
| Repeat  steps |  | 34 cycles |
| Extend  primers | 72 | 8 min |
| Hold | 4 | Forever |

[1. Steen, C. B., Liu, C. L., Alizadeh, A. A. & Newman, A. M. Profiling Cell Type Abundance and Expression in Bulk Tissues with CIBERSORTx. *Methods Mol. Biol. Clifton NJ* **2117**, 135–157 (2020).](https://www.zotero.org/google-docs/?JugEbK)

[2. Bankhead, P. *et al.* QuPath: Open source software for digital pathology image analysis. *Sci. Rep.* **7**, 16878 (2017).](https://www.zotero.org/google-docs/?JugEbK)

[3. Armbruster, D. A. & Pry, T. Limit of Blank, Limit of Detection and Limit of Quantitation. *Clin. Biochem. Rev.* **29**, S49–S52 (2008).](https://www.zotero.org/google-docs/?JugEbK)

[4. Berben, L. *et al.* Computerised scoring protocol for identification and quantification of different immune cell populations in breast tumour regions by the use of QuPath software. *Histopathology* **77**, 79–91 (2020).](https://www.zotero.org/google-docs/?JugEbK)
