# Supplementary material for: Gorham-Stout case report: a multi-omic analysis reveals recurrent fusions as new potential drivers of the disease
Source: BMC Med Genomics. 2022 Jun 6;15:128. doi: 10.1186/s12920-022-01277-x (PMC9169400; doi:10.1186/s12920-022-01277-x)

Supplementary gel photograph. The PCR product was amplified from normal and Grahm Stout(GS) tissue. The following PCR products were run in the Gel1 electrophoresis. 1.Control ATG101-SLC4A8, **2**.GS- ATG101-SLC4A8, **3**.Negative PCR Control (ATG101-SLC4A8); **4**.Control PPTC7-AC007091, **5**.GS PPTC7-AC007091, **6.**Negative PCR Control (PPTC7-AC007091), **7**.Control GRIP1-AC113346.2 **8.**GS- GRIP1-AC113346.2 **9.**Negative PCR Control (GRIP1-AC113346.2) **10**.Control SGCD-DNAH11, **11.**GS-SGCD-DNAH11, **12.**Negative PCR Control(GS-SGCD-DNAH11), **13**.Control SLC48-ATP5MC2, **14.**GS- SLC48-ATP5MC2, **15.**Negative PCR Control(SLC48-ATP5MC2), 16.Control HLAF-HLAH, **17.**GS- HLAF-HLAH, **18.**Negative PCR Control(HLAF-HLAH). In the second gel(Gel2) only one set of amplified products were used in the final photograph as **19**.Control FRS2-AC004485.1, **20.**GS- FRS2-AC004485.1 **21.**Negative PCR Control(FRS2-AC004485.1). The wells 19, 20 and 21 were cropped in the final photgraph including the PCR marker. A quick load PCR marker was used from NEB #N0475.

1 2 3 4 5 6 7 8 9 10 11 12 13 14 15 16 17 18 Marker

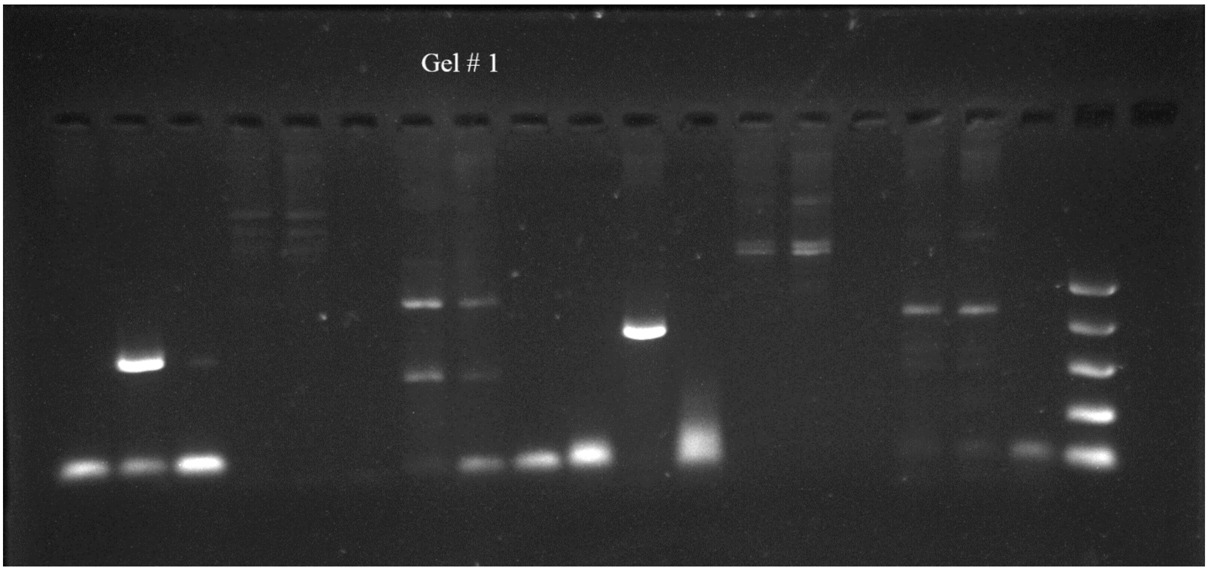

19 20 21

Marker

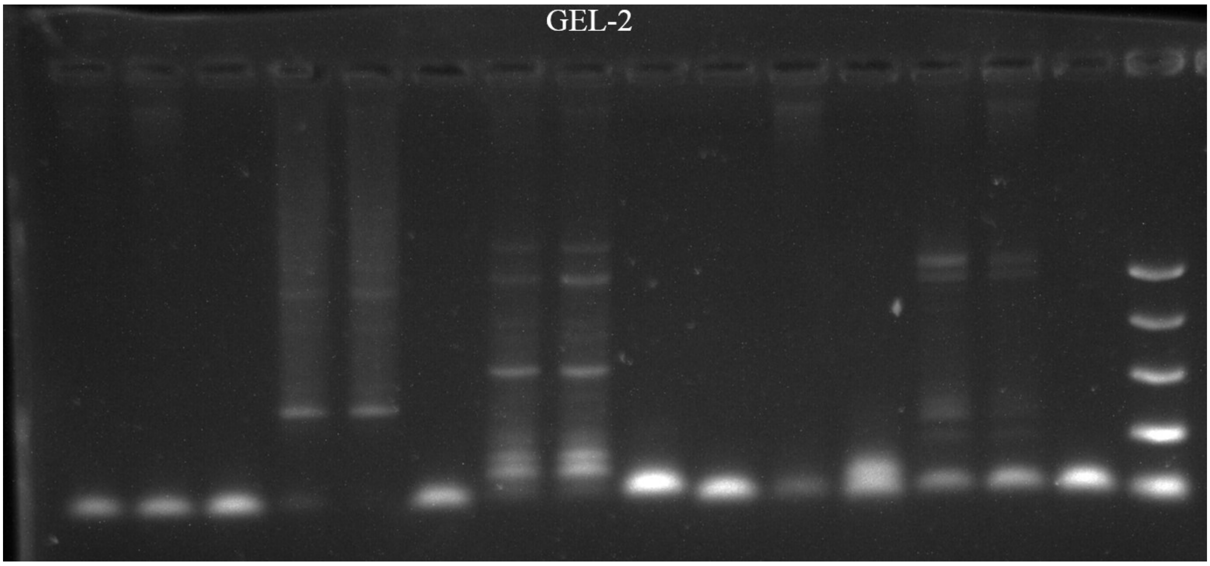

Supplement: Supplementary file 4 — Additional file4: Figure S2. Gel electrophoresis containing control, Gorham-Stout tissue, and negative control of the gene fusion candidates. In the second gel only one set of amplified products was used in the final photograph as 19.Control FRS2-AC004485.1, 20.GS-FRS2-AC004485.1 21.Negative PCR Control (FRS2-AC004485.1). The wells 19, 20, and 21 were cropped in the final photograph including the PCR marker. A quick load PCR marker was used from NEB #N0475. [file 12920_2022_1277_MOESM4_ESM.pdf]
