# Supplementary material for: Gorham-Stout case report: a multi-omic analysis reveals recurrent fusions as new potential drivers of the disease
Source: BMC Med Genomics. 2022 Jun 6;15:128. doi: 10.1186/s12920-022-01277-x (PMC9169400; doi:10.1186/s12920-022-01277-x)
Supplement: Supplementary file 7 — Additional file7: Figure S3. Barplot of RNA-seq expression (TPM) of VEGFC/D and NOTCH 2/3/4 genes in Gorham-Stout tissue compared with adjacent normal. [file 12920_2022_1277_MOESM7_ESM.pdf]

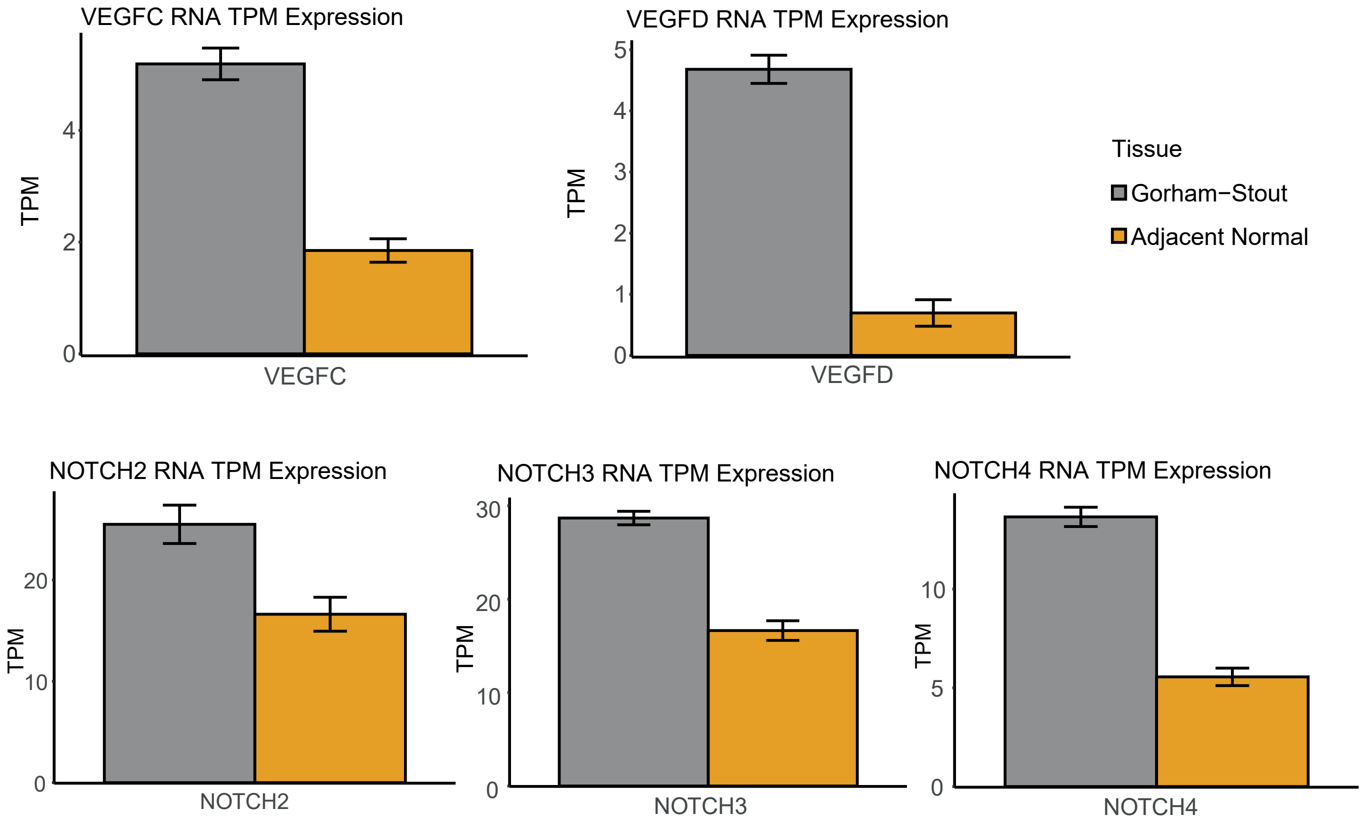

Supplementary Figure 3. Barplot of RNA-seq expression (TPM) of VEGFC/D and NOTCH 2/3/4 genes in Gorham-Stout tissue compared with adjacent normal.
