# Supplementary material for: Gorham-Stout case report: a multi-omic analysis reveals recurrent fusions as new potential drivers of the disease
Source: BMC Med Genomics. 2022 Jun 6;15:128. doi: 10.1186/s12920-022-01277-x (PMC9169400; doi:10.1186/s12920-022-01277-x)
Supplement: Supplementary file 8 — Additional file8: Figure S4. Gene fusion between ATG101 and SLC4A8 detected in Gorham-Stout patient. RNA evidence reads: 118 encompasing and 138 spanning reads. DNA evidence reads: 411 encompassing and 139 spanning reads in Gorham-Stout tissue. 0 reads found in normal tissue. [file 12920_2022_1277_MOESM8_ESM.pdf]

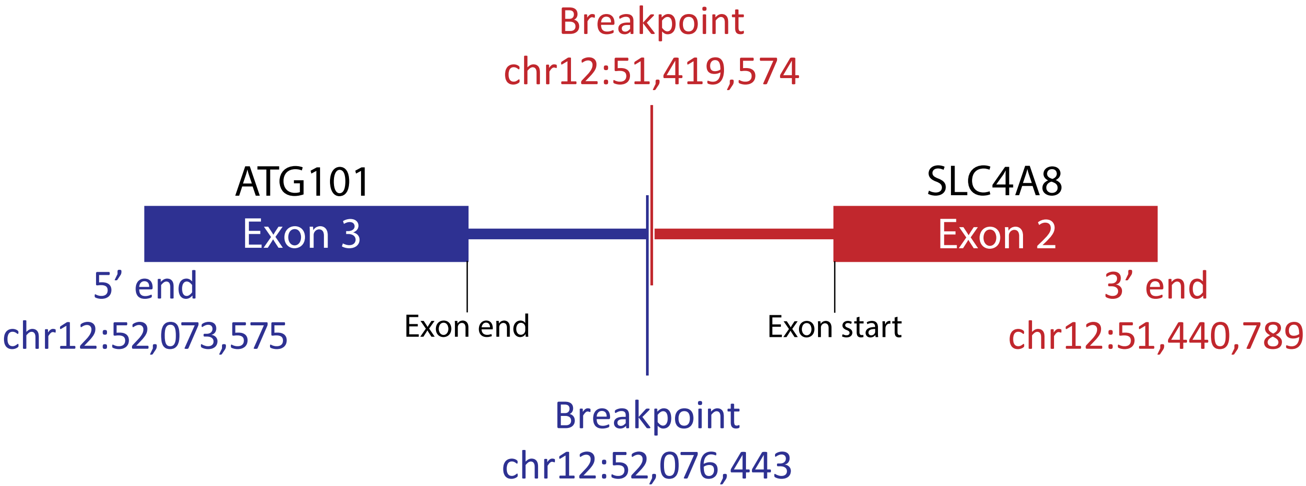

Supplementary Figure 4. Gene fusion between ATG101 and SLC4A8 detected in Gorham-Stout patient. RNA evidence reads: 118 encompasing and 138 spanning reads. DNA evidence reads: 411 encompassing and 139 spanning reads in Gorham-Stout tissue. 0 reads found in normal tissue.
